# Group B *Streptococcus* Serotype III Sequence Type 283 Bacteremia Associated with Consumption of Raw Fish, Singapore

**DOI:** 10.3201/eid2211.160210

**Published:** 2016-11

**Authors:** Shermin Tan, Yijun Lin, Kelly Foo, Han Fang Koh, Charlene Tow, Yiwen Zhang, Li Wei Ang, Lin Cui, Hishamuddin Badaruddin, Peng Lim Ooi, Raymond Tzer Pin Lin, Jeffery Cutter

**Affiliations:** Ministry of Health, Singapore

**Keywords:** group B *Streptococcus*, streptococci, Streptococcus agalactiae, sequence type 283, serotype III, bacteria, outbreak, raw fish, yusheng, raw foods, food-borne infections, food safety, Singapore

## Abstract

We conducted a retrospective study of 40 case-patients and 58 controls as part of a nationwide investigation of a group B *Streptococcus* outbreak in Singapore in 2015. Eating a Chinese-style raw fish dish (yusheng) was a major risk factor for bacteremia, particularly caused by serotype III sequence type 283.

Group B *Streptococcus* (GBS) disease is caused by *Streptoccocus agalactiae*, a common commensal bacteria found in the gastrointestinal and genital tracts of 15%–30% of healthy adults (*1*). GBS can cause invasive infections, especially in elderly persons and those who have underlying medical conditions, such as diabetes mellitus (*2*). The serotype III sequence type 283 (ST283) strain has been associated with invasive disease in adults (*3*). GBS infection is not a reportable disease in Singapore, and positive culture isolates are not routinely serotyped.

GBS can also cause bovine mastitis (*4*) and disease in saltwater and freshwater fish (*5*–*7*). GBS serotypes Ia and III ST283 and ST491 have also been detected in tilapia from Southeast Asia (*8*).

In June 2015, the Ministry of Health (MOH) in Singapore was alerted to an increase in the number of GBS bacteremia cases among adults admitted to 6 acute-care public hospitals. Seven acute-care public hospitals in Singapore account for >80% of beds for acute-care patients; no increase in cases was observed at a women’s and children’s hospital (Figure).

**Figure:**
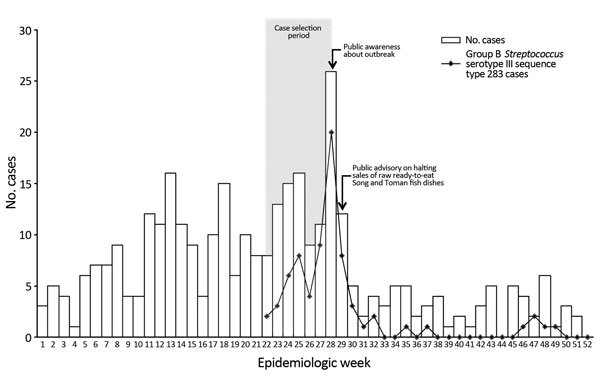
Timeline of group B *Streptococcus* bacteremia cases reported in 6 public hospitals, Singapore, epidemiologic weeks 1−52, 2015.

Anecdotal links between eating raw fish and illness were reported for some patients. Although 1 study in the United States found a link between eating fish and risk for GBS colonization in adults (*9*), and fish and humans can be infected with the same strains (*5**,**8*), links between eating fish and GBS infection in humans have not been established. To identify risk factors associated with this purportedly foodborne outbreak, the MOH conducted a case−control study in July 2015 as part of a national outbreak investigation.

## The Study

The retrospective case−control study comprised 40 case-patients and 58 healthy contacts. Case-patients were defined as patients from the 6 hospitals who had GBS isolated from blood cultures during June 1−July 14, 2015, the period of highest GBS bacteremia incidence. Healthy household members or colleagues of case-patients were selected as controls because they were more likely to have had similar dietary patterns.

Using a standardized interviewer-administered questionnaire, we obtained demographic data, medical history, and 2-week food and exposure histories. Information was also obtained on consumption of raw fish, seafood, or unpasteurized dairy products; exposure to live fish; or invasive medical procedures. Data were collected over a period of 1 week.

Positive blood culture isolates from 36 of the 40 case-patients were obtained from hospital laboratories. Capsular typing was conducted by the National Public Health Laboratory (NPHL) of the MOH by using a PCR-based method (*10*). The NPHL was unable to obtain positive blood culture isolates from 4 case-patients for further typing. After the case selection period, the public hospitals were requested to report all cases of GBS bacteremia to the MOH and submit positive blood culture isolates for capsular typing by the NPHL. Sequence types were determined by using multilocus sequence typing (*11**,**12*).

Mean age of case-patients (61.5 years) was higher than that of controls (44.8 years) (Table 1). A larger proportion of case-patients (52.5%) were male than controls (31.0%). Most case-patients and controls were of Chinese ethnicity; none had occupational exposure to live animals. A total of 72.5% of case-patients reported underlying medical conditions, more than twice the total for controls (31.0%).

**Table 1:** Characteristics of case-patients and controls in group B *Streptococcus* outbreak investigation, Singapore, 2015*

Characteristic	No. (%) case-patients, n = 40	No. (%) controls, n = 58	p value
Age group, y			<0.0005
0–4	0	0	
5–14	0	1 (1.7)	
15–24	0	5 (8.6)	
25–34	0	9 (15.5)	
35–44	6 (15.0)	13 (22.4)	
45–54	8 (20.0)	9 (15.5)	
55–64	7 (17.5)	11 (19.0)	
>65	19 (47.5)	5 (8.6)	
Unknown	0	5 (8.6)	
Mean age, y	61.5	44.8	
Median age, y	61.5	44.0	
Age range, y	33–68	14–95	
Sex			0.038
M	21 (52.5)	18 (31.0)	
F	19 (47.5)	40 (69.0)	
Ethnicity			0.527
Chinese	36 (90.0)	47 (81.0)	
Malay	3 (7.5)	6 (10.3)	
Indian	0	2 (3.4)	
Other	1 (2.5)	3 (5.2)	
Housing type†	7 (17.5)	10 (17.2)	0.991
3-room flats and smaller‡	17 (42.5)	22 (37.9)	
4-room HDB flats	8 (20.0)	13 (22.4)	
Executive/5-room HDB flats	4 (10.0)	7 (12.1)	
Private housing	4 (10.0)	6 (10.3)	
Concurrent illness			<0.0005
No	11 (27.5)	40 (69.0)	
Yes	29 (72.5)	18 (31.0)	
Diabetes mellitus	15 (37.5)	5 (8.6)	0.001
Cancer	3 (7.5)	0	0.065
Cardiovascular disease	22 (55.0)	13 (22.4)	0.001
Renal disease	5 (12.5)	0	0.010
Liver disease	3 (7.5)	0	0.065
Gastrointestinal disease	1 (2.5)	0	0.408
Respiratory disease	3 (7.5)	0	0.065
Blood disease	2 (5.0)	1 (1.7)	0.565

*HDB, Housing Development Board. †Housing type was used as a proxy for socioeconomic status; >80% of the population in Singapore live in public housing managed by the HDB. ‡HDB flats are considered public housing by the HDB.

Univariable logistic regression analysis showed that eating raw fish was associated with GBS bacteremia (Table 2). Eating yusheng, Chinese-style raw fish dish usually prepared with freshwater fish, such as Asian bighead carp or snakehead fish, or saltwater fish, such as wolf herring, was a major risk factor (odds ratio [OR] 5.11, 95% CI, 1.93–13.52). The odds of GBS bacteremia among case-patients >65 years of age were 17.73 times that of those 14–44 years of age (95% CI 4.73–66.52). We found no association between patient age and eating yusheng or between GBS and other exposures. However, diabetes mellitus (OR 6.36, 95% CI 2.08–19.45) and cardiovascular disease (OR 4.23, 95% CI 1.76–10.17) were major risk factors.

**Table 2:** Association between bacteremia and risk factors in a case−control study of a group B *Streptococcus* outbreak, Singapore, 2015*

Risk factor	No. (%) case-patients, n = 40	No. (%) controls, n = 58	Univariable model			Multivariable model†	
			Crude OR (95% CI)	p value		Adjusted OR (95% CI)†	p value
Raw fish	19 (47.5)	11 (19.0)	3.87 (1.57–9.54)	0.003		8.58 (2.25–32.69)	0.002
Yusheng‡	18 (45.0)	8 (13.8)	5.11 (1.93–13.52)	0.001		11.38 (2.76–46.98)	0.001
Sashimi/sushi	5 (12.5)	3 (5.2)	2.62 (0.59–11.65)	0.206		4.39 (0.74–25.91)	0.103
Raw shellfish	1 (2.5)	1 (1.7)	1.46 (0.09–24.07)	0.791		5.18 (0.23–114.95)	0.299
Fish-related activities§	2 (5.0)	6 (10.3)	0.46 (0.09–2.39)	0.352		0.71 (0.10–5.07)	0.736

*OR, odds ratio.  †Included age group (14–44, 45–54, 55–64, and >65 y), sex, and medical conditions as independent variables in a logistic regression model. ‡Chinese-style raw fish dish. §Included fishing, fish spas, fish rearing, and gutting of fish.

A multivariable model that controlled for age group, sex, and concurrent medical conditions showed that the odds of GBS bacteremia among those who had eaten raw fish were 8.58 times that of those who had not eaten raw fish (95% CI 2.25–32.69). Eating yusheng remained a major risk factor in separate analysis by type of raw fish food items consumed (adjusted OR 11.38, 95% CI 2.76–46.98) (Table 2).

Mean and median durations between eating raw fish and onset of illness were 3.5 and 4.5 days, respectively. Mean and median durations between eating yusheng and onset of illness were 3.7 and 4.0 days, respectively.

Of 36 isolates tested, all from case-patients who ate raw fish (n = 19) were serotype III ST283. Isolates from case-patients who did not eat raw fish had various serotypes: Ia (n = 1), II (n = 4), III (n = 7), VI (n = 4), and VII (n = 1). Restricted analysis of 25 case-patients with serotype III ST283 bacteremia and 58 controls showed a strong association between eating yusheng and serotype III ST283 bacteremia (adjusted OR 25.92, 95% CI 5.38–124.96).

Mean age of case-patients infected with ST283 was significantly younger than patients infected with non-ST283 (57.4 vs. 68.6 years; p = 0.014). A significantly lower proportion of patients infected with ST283 than non-ST283 reported having underlying medical conditions (56% vs. 100%; p = 0.003).

## Conclusions

We found a strong association between eating yusheng and GBS serotype III ST283 bacteremia. These findings are further supported by the presence of GBS serotype III ST283 in fish samples tested by the Agri-Food and Veterinary Authority of Singapore and the National Environment Agency (NEA) (*13*). Other major risk factors for GBS bacteremia, such as age >65 years, diabetes mellitus, and cardiovascular disease, were consistent with known risk factors.

After a public advisory was issued on July 24, 2015, to halt sales of raw fish dishes made with Asian bighead carp and snakehead fish (*14*), the weekly number of invasive GBS cases decreased rapidly in <2 weeks, from a peak of 26 cases/week to an average of <5 cases/week. This decrease supported the hypothesis of a short incubation period after eating raw fish. The proportion of serotype III ST238 cases also decreased rapidly after the advisory (Figure).

This study might be limited by recall bias, in which case-patients were more likely to recall exposure to raw fish, given the increased public awareness at the time of the study. Not all patients given a diagnosis within the case selection period were interviewed because some were not available or too ill to be interviewed. We obtained a case:control ratio of only 1:1.45 because of difficulties in recruiting participants who were willing to be interviewed. Case-patients detected before June 1 were not recruited because they would have been unlikely to provide an accurate food history. Nonetheless, we still obtained statistical power >80% in detecting a 4-fold increased odds of GBS bacteremia after eating yusheng. Although we found no strong association between GBS bacteremia and consumption of sashimi, sushi, or raw shellfish or exposure to fish-related activities, power to detect statistical significance was limited because of small sample sizes.

Although we found a strong epidemiologic link between eating raw fish, particularly yusheng, and GBS serotype III ST283 bacteremia, further studies are needed to understand the precise mechanism of transmission from raw fish to humans and the pathologic process that takes place for infection to occur. In the interest of public health, the NEA has since banned use of freshwater fish in all ready-to-eat raw fish dishes sold by retail food establishments (*15*). The NEA and the Agri-Food and Veterinary Authority of Singapore have also increased measures to ensure that raw saltwater fish intended for consumption are closely monitored and tested for safety.
